# Pharmacological and Genetic Inhibition of PD-1 Demonstrate an Important Role of PD-1 in Ischemia-Induced Skeletal Muscle Inflammation, Oxidative Stress, and Angiogenesis

**DOI:** 10.3389/fimmu.2021.586429

**Published:** 2021-03-19

**Authors:** Xiaoguang Liu, Xinyu Weng, Weihua Xiao, Xin Xu, Yingjie Chen, Peijie Chen

**Affiliations:** ^1^ College of Sports and Health, Guangzhou Sport University, Guangzhou, China; ^2^ Lillehei Heart Institute and Cardiovascular Division, University of Minnesota Medical School, Minneapolis, MN, United States; ^3^ Department of Cardiology, Zhongshan Hospital, Shanghai Institute of Cardiovascular Diseases, Fudan University, Shanghai, China; ^4^ School of Kinesiology, Shanghai University of Sport, Shanghai, China; ^5^ Department of Physiology and Biophysics, University of Mississippi Medical Center, Jackson, MS, United States

**Keywords:** PD-1, angiogenesis, inflammation, oxidative stress, hindlimb ischemia

## Abstract

Angiogenesis is an important process under both physiological and pathophysiological conditions. Here we investigated the role and the underlying mechanism of PD-1 in hindlimb ischemia-induced inflammation and angiogenesis in mice. We found that inhibition of PD-1 by genetic PD-1 knockout or pharmacological PD-1 blocking antibodies dramatically attenuated hindlimb blood perfusion, angiogenesis, and exercise capacity in mice after femoral artery ligation. Mechanistically, we found that PD-1 knockout significantly exacerbated ischemia-induced muscle oxidative stress, leukocyte infiltration and IFN-γ production before abnormal angiogenesis in these mice. In addition, we found that the percentages of IFN-γ positive macrophages and CD8 T cells were significantly increased in P-1 knockout mice after hindlimb ischemia. Macrophages were the major leukocyte subset infiltrated in skeletal muscle, which were responsible for the enhanced muscle leukocyte-derived IFN-γ production in PD-1 knockout mice after hindlimb ischemia. Moreover, we demonstrated that IFN-γ significantly attenuated vascular endothelial cell proliferation, tube formation and migration *in vitro*. IFN-γ also significantly enhanced vascular endothelial cell apoptosis. In addition, the total number of TNF-α positive leukocytes/muscle weight were significantly increased in PD-1^-/-^ mice after hindlimb ischemia. These data indicate that PD-1 exerts an important role in ischemia-induced muscle inflammation and angiogenesis.

## Introduction

Angiogenesis is an important process in maintaining tissue homeostasis under various physiological and pathophysiological conditions such as reproduction, embryonic development, injury repair, and tumor growth. Angiogenesis after ischemia is essential for the restoration of blood flow to ischemic limbs, brain and heart through regeneration of both micro-vessels and collateral vessels (1). Abnormal angiogenesis contributes to pathophysiological processes in coronary artery disease, peripheral vascular disease, leg ulceration, cancers, and diabetic retinopathy etc. (2, 3). Reduced angiogenesis and blood perfusion in skeletal muscle also attenuate muscle function or exercise capacity. Therefore, investigating the molecular mechanism of angiogenesis may advance our understanding of the development and progression of clinical diseases related to abnormal angiogenesis.

While the primary mechanism of ischemia-induced angiogenesis involves the production of hypoxia-inducible factor-1α (HIF-1 α) protein, and the consequent increase of expression of vascular endothelial growth factor (VEGF) (4), it has been shown that immune system also plays a pivotal role in modulating ischemia-induced hindlimb angiogenesis (5–8). Thus, studies have demonstrated that ischemia causes increased cytokine production and leukocyte infiltration in ischemic tissues (5, 6, 9, 10). Leukocyte subsets such as macrophages, CD4+ T cells, CD8+ T cells, and regulatory T cells (Tregs) regulate ischemia-induced angiogenesis (11–13). Many cytokines and chemokines such as IL-10 also affect hindlimb ischemia-induced inflammation and angiogenesis (6, 14, 15).

Programmed cell death protein 1 (PD-1), also known as cluster of differentiation 279 (CD279), is a cell membrane protein that expressed on T cells. PD-1’s ligands are PD-L1 and PD-L2 that are expressed on the surface of dendritic cells, macrophages, and some tumor cells. PD-1 plays an important role in immune tolerance to attenuate autoimmune diseases through inhibiting both the induction and the maintenance of T cell tolerance (16–18). The binding of PD-1 to its ligands on tumor cells suppresses the T cell-induced killing of cancer cells. Thus, therapeutic antibodies that block the interaction between PD-1 and its ligands are used for cancer treatments (19–23). However, the immune checkpoint inhibitors against the binding of PD-1 with its ligands also result in moderate to severe tissue toxicities in various organs such as lung, skin, small intestine, colon, kidney, liver, pancreas, joints, heart, and vascular system in cancer patients (24–26). Inhibition of PD-1 or its ligands also exacerbates atherosclerosis, cardiac inflammation and cardiac dysfunction (25, 26). However, the underlying molecular mechanism (s) of PD-1 inhibition on tissue toxicities is not totally clear.

Since PD-1 regulates tissue inflammatory responses, we postulated that inhibition of PD-1 might attenuate ischemia-induced angiogenesis through exacerbating the inflammatory response, a pathological alteration that could contribute to tissue toxicities by PD-1 inhibitors. Consequently, we investigated the role and the underlying mechanism of inhibition of PD-1 by genetic PD-1 gene deletion (PD-1^-/-^) or pharmacological PD-1 blocking antibodies (mAbs) in hindlimb ischemia-induced muscle inflammation, angiogenesis, and injury repair in mice. The data indicate that PD-1 exerts an important role in regulating muscle inflammation, angiogenesis, and blood perfusion, as well as the overall exercise capacity in mice after hindlimb ischemia.

## Materials and Methods

Detailed methods are available in the **Supplemental Materials**.

### Animals

**Figure 1:**
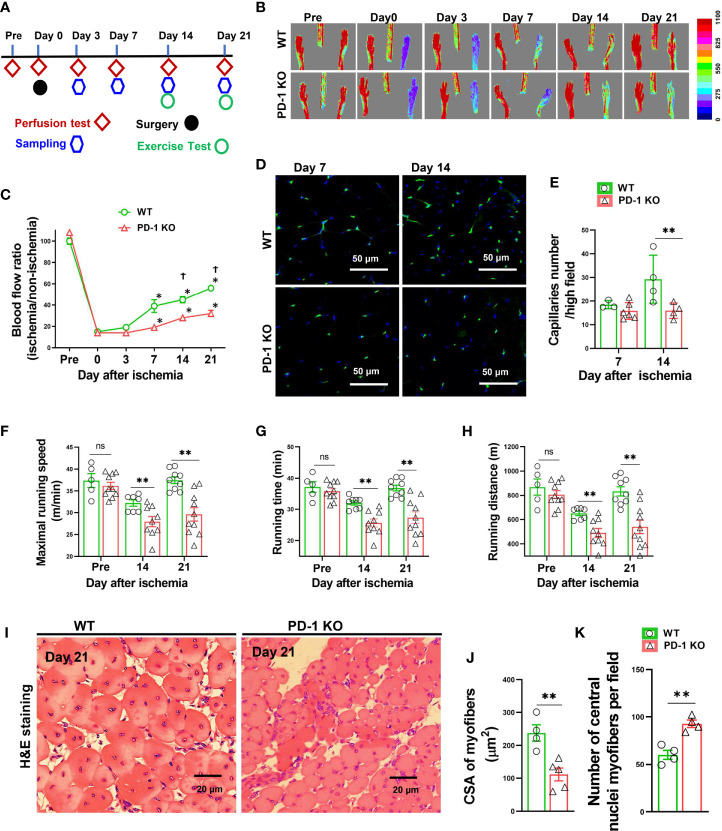
PD-1^-/-^ impaired blood perfusion, angiogenesis, exercise capacity and skeletal muscle regeneration in mice after hindlimb ischemia. **(A)** The diagram shows the time of relevant interventions. **(B)** Laser doppler perfusion imaging of ischemic and non-ischemic hind limb as indicated. **(C)** Quantification of hind limb perfusion in WT and PD-1**^-/-^** mice (percentage of perfusion relative to the nonischemic limb). *P < 0.05 VS Day 0. ^†^P < 0.05 VS PD-1**^-/-^** mice. n=5-9. **(D)** Detecting calf muscle micro-vessel densities by staining with Isolectin GS-IB4 (IB4) at day 7 and 14 after hindlimb ischemia. **(E)** Quantified data of micro-vessel number per high field in WT and PD-1^-/-^ mice at day 7 and 14 after ischemia. n=3-6. **(F-H)** Quantitative analysis of maximal speed, maximal running time, and maximal running distance when reaching maximal speed. n=5-10. **(I-K)** Representative images of H&E staining, quantified myocyte fiber cross-sectional area (CSA) and number of muscle fibers with central nuclei in WT and PD-1**^-/-^** mice 21 day after hindlimb ischemia. n=4-5. CSA of each sample was the average of at least 60 myocytes. ANOVA followed by a Bonferroni correction post-hoc test was used to test for differences among more than 2 groups. Two groups were compared *via* Student’s t-test. *P < 0.05 between corresponding groups. **P < 0.01 between corresponding groups. ns, not significant.

PD-1**^-/-^** mice and corresponding wild type (WT) mice of C57BL/6J background were purchased from Jackson Laboratory. Female mice at an age of 8 weeks were used for hindlimb ischemia surgery as illustrated in **Figure 1A**. InVivoPlus PD-1 antibodies (Bio Xcell, Clone: J43) were administered at a dose of 200 μg/mouse as illustrated in **Figure 1A**. The investigation conforms with the Guide for the Care and Use of Laboratory Animals published by the US National Institutes of Health (NIH Publication NO. 85-23, revised 1996). The study was approved by the Animal Care and Use Committee of Shanghai University of Sport, China.

*Mouse euthanasia was performed by exsanguination after anesthesia with isoflurane.*

### Hindlimb Ischemia Model and Laser-Doppler Perfusion Imaging

Mice were given buprenorphine-SR at a dose of 2mg/kg one hour before surgery. Mice were anesthetized using isoflurane (isoflurane at 3% for induction and 2% for maintenance), and the hairs on both hindlimbs were shaved with a small animal electric trimmer (Shenzhen Ruiwode Life Technology, Shezhen, China). The femoral artery (FA) was exposed by incision of the skin at the middle portion of the left hindlimb. Both proximal end and distal end of the FA were ligated and the vessel between the ligatures was excised. The skin incision was then closed with 5-O Vicryl sutures.

Shortly after the surgery, as well as 3, 7, 14, and 21days after surgery, anesthetized mice were placed on heating pads for several minutes and the blood perfusions of both hindlimbs were determined with a high-resolution laser doppler imager (MOORLDI2-HIR system, Moor instruments, UK). Color-coded images were obtained with a Moor laser-Doppler imager (LDI) scanner. The perfusions of each mouse were calculated by the Moor LDI software at regions of interest. The perfusion of the ischemia limb is expressed as a percentage of the perfusion to the non-ischemic limb.

### Determination of Mouse Exercise Capacity

Mouse exercise capacity on the treadmill was tested. Detailed methods are available in the **Supplemental Materials**.

### Histological Staining

H&E staining, and Masson’s Trichrome staining were used for detecting muscle fiber size and fibrosis. The capillary density was determined by staining of Isolectin GS-IB4 (IB4) using Alexa Fluor™ 488 Conjugate antibodies (Invitrogen, California, United States). Immune staining of CD45 was used for detecting leukocytes.

Intracellular reactive oxidative species (ROS) were determined by dihydroethidium staining kit (Invitrogen, California, United States) according the manufacturer’s instructions. Briefly, gastrocnemius muscle was immediately embedded in an Optimum Cutting Temperature compound and stored at -80°C. Dihydroethidium (DHE) (5μmol/L) was applied to 10μm fresh frozen tissue sections, incubated in the light-protected humidified chamber at 37°C for 30min, washed with PBS three times, 5min per wash, and the sections were then cover-slipped using Mounting Medium (Abcam, ab104139).

### RNA Extraction and Real-Time PCR

Total RNA samples were extracted from gastrocnemius muscle using TRIzol (Invitrogen, California, United States). RNA samples were then reverse-transcribed into cDNA using a kit from Thermo Scientific (Revertaid™ First Strand cDNA Synthesis Kit), followed by semi-quantitative real-time PCR using SYBR Green/ROX qPCR Master mix (Thermo Scientific, Massachusetts, United States). Data were calculated by the ΔΔCT method. The primers used in this experiment are listed in **Supplemental Table S1**.

### Enzyme-Linked Immunosorbent Assay (ELISA)

Gastrocnemius muscle IFN-γ levels were measured using a mouse IFN-γ ELISA kit from Invitrogen (cat: BMS606) according the manufacturer’s instructions.

### Detection of Leukocyte Intracellular Cytokine Production

Tissues were digested in PBS buffer with collagenase II (2 mg/ml, Invitrogen) and DNase I (150 Ug/ml, Sigma) for 40 min. To enrich the leukocytes from the digested muscle, the isolated cells were resuspended in 40% Percoll (Sigma-Aldrich) overlaid on 80% Percoll, then centrifuged at 400 g for 25 min. The interphase that contains leukocytes was collected. The leukocytes were then stimulated with Cell Stimulation Cocktail (Phorbol 12-Myristate 13-Acetate (40.5 µM), lonomycin (670 µM), Brefedin A (5.3 mM), Monensin (1 mM) in Ethanol (500X)) (Invitrogen, California, United States) for 5 h at 37°C in completed RPMI-1640 media (Invitrogen, California, United States). Cell suspensions were stained with antibodies, then processed detecting the corresponding leukocyte subsets and cytokine productions using a flow cytometer (BD LSRFortessa™ X-20, United States). Data were analyzed by FlowJo software version 10 (FlowJo LLC, United States).

### HUVEC Cell Proliferation, Tube Formation, Migration and Apoptosis Assay

Primary Human umbilical vein endothelial cells (HUVEC) (LONZA, Basel, Switzerland) at passage 4 to 6 were used for the experiments. HUVEC cell proliferation was assayed by CyQUANT^®^ Cell Proliferation Assay Kit (Invitrogen, California, United States) after IFN-γ (Biolegend, San Diego, United States) (100U/ml) or vehicle (0.1% bovine serum albumin (BSA) treatment for 24 hours. Tube formation capacity was determined using an *in vitro* angiogenesis assay kit from R&D Systems (Minneapolis, United States). HUVEC migration capacity were assayed by scratch test. Cell apoptosis was determined using FITC Annexin V Apoptosis Detection Kit with 7-aminoactinomycin D (7-AAD) (Biolegend, San Diego, United States).

### Data Analysis

Data were presented as mean ± standard error. Two-way ANOVA followed by a Bonferroni correction post-hoc test was used to test for differences among more than 2 groups. Two groups were compared *via* Student’s t-test by GraphPad Prism 8 software. All pairwise p-values are two-sided. The null hypothesis was rejected at p < 0.05.

## Results

### PD-1^-/-^ Impaired Hindlimb Blood Perfusion and Micro-Vessel Regeneration in Mice After Ischemia

To study the role of PD-1 in angiogenesis development, the relative hindlimb blood perfusions were determined in WT and PD-1^-/-^ mice before surgery, shortly after hindlimb ischemia, and 3, 7, 14, and 21 days after hindlimb ischemia as illustrated in **Figure 1A**. As presented in **Figures 1B, C**, the ischemia procedure caused ~80% decrease of hindlimb blood perfusion in WT mice shortly after surgery, and 3 days after hindlimb ischemia, and the blood perfusion was significantly recovered 7, 14 and 21 days after hindlimb ischemia. As compared with WT mice, hindlimb ischemia caused a similar decrease of blood perfusion in PD-1^-/-^ mice shortly after surgery and 3 days after hindlimb ischemia. However, the hindlimb blood perfusion had recovered significantly less in PD-1^-/-^ mice 14 days after hindlimb ischemia as compared with corresponding mice (45 ± 3% in WT vs 30 ± 1% in PD-1^-/-^ mice, p<0.05), and 21 days (56 ± 1% in WT mice vs 39 ± 3% in PD-1^-/-^ mice, p<0.05) (**Figures 1B, C**).

To understand whether the reduced blood perfusion in PD-1^-/-^ mice had resulted from the differences in micro-vessel regeneration, we further determined micro-vessel density in PD-1^-/-^ mice and WT mice after ischemia. The results show that muscle micro-vessel densities were comparable between PD-1^-/-^ mice and WT mice 7 days after femoral artery ligation, while the vessel density was significantly lower in PD-1^-/-^ mice as compared with WT mice 14 days (27.4 ± 1.74 in WT mice vs 15.89 ± 1.62 in PD-1^-/-^ mice, P<0.05) after hindlimb ischemia (**Figures 1D, E**), indicating PD-1^-/-^ impaired micro-vessel regeneration.

### PD-1^-/-^ Severely Impaired Mouse Exercise Capacity and Muscle Fiber Repair After Hindlimb Ischemia

Since the hindlimb perfusion regulates the exercise capacity, to further objectively evaluate the hindlimb perfusion requirement, we determined the mouse exercise capacity before ischemia, as well as 14 and 21 days after hindlimb ischemia (27). We found that the exercise capacities were not different between WT and PD-1^-/-^ mice under control conditions, but the exercise capacities of PD-1^-/-^ mice were significantly decreased as compared with WT mice at 14 and 21 days after femoral artery ligation, as evidenced by significant decreases of maximal running speed, duration of running, and maximal running distance in PD-1^-/-^ mice (**Figure 1F-H**). In addition, muscle fiber cross-sectional areas were significantly decreased in PD-1^-/-^ mice as compared to WT mice 21 days after hindlimb ischemia (**Figures 1I, J**). Moreover, the number of muscle fibers with central nuclei, a pathological marker for abnormal muscle differentiation, was significantly increased in PD-1^-/-^ mice as compared to WT mice after hindlimb ischemia (**Figure 1K**). PD-1^-/-^ mice also had a significant increase of muscle fibrosis (2.5-fold) 21 days after hindlimb ischemia (**Supplemental Figure S2A, B**).

### PD-1^-/-^ Exacerbated the Accumulation of Leukocytes, Macrophages, T Cells and NK Cells in Mouse Calf Muscle After Hindlimb Ischemia

**Figure 2:**
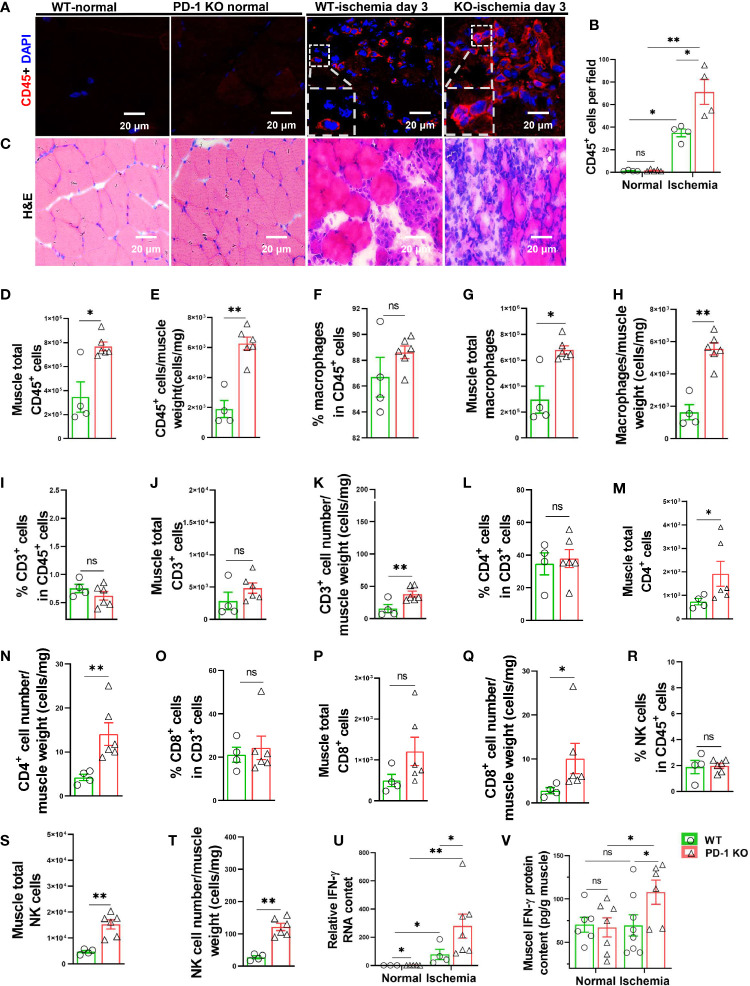
PD-1^-/-^ exacerbated calf muscle inflammation in mice after hindlimb ischemia. **(A)** Representative images of muscle CD45^+^ cells (red). **(B)** Quantification of muscle CD45^+^ cells by immunofluorescence. n=4-5. **(C)** Representative images of H&E from WT and PD-1^-/-^ mice undergoing ischemia or no-ischemia surgery. n=4-5. **(D, E)** Quantification of CD45^+^ cells by flow cytometry. n=4-6. **(F-H)** Quantification of macrophage (F4/80^+^ CD11b^+^) by flow cytometry. n=4-6. **(I-K)** Quantification of CD3^+^ T cells by flow cytometry. n=4-6. **(L-N)** Quantification by flow cytometry of CD4+ cells (CD45^+^ CD3^+^ CD4^+^). n=4-6. **(O-Q)** Quantification by flow cytometry of CD8+ cells (CD45^+^ CD3^+^ CD8^+^). n=4-6. **(R-T)** Quantification by flow cytometry of NK cells (CD45^+^ CD3^-^ NK1.1^+^). n=4-6. **(U)** Quantitative reverse-transcriptase polymerase chain reaction (RT-PCR) results of IFN-γ in skeletal muscle. n=4-7. **(V)** Quantitative ELISA results of IFN-γ in skeletal muscle of mice with ischemia or non-ischemia. n=6-8. ANOVA followed by a Bonferroni correction post-hoc test was used to test for differences among more than 2 groups. Two groups were compared *via* Student’s t-test. *P < 0.05 between corresponding groups. **P < 0.01 between corresponding groups. ns, not significant.

Since hindlimb ischemia often causes apparent muscle inflammation 3 to 7 days after femoral artery ligation (11, 28), and since the blood perfusion was largely unchanged until 14 days after the initial femoral artery ligation, we reasoned that PD-1 might attenuate ischemia-induced angiogenesis by exacerbating pro-inflammatory cytokine production and muscle inflammation. Consequently, we determined the inflammation in PD-1^-/-^ and WT mice 3 days after femoral artery ligation. Leukocytes are almost undetectable in normal calf muscles obtained from either PD-1^-/-^ or WT mice (**Figure 2A-C**). However, immunological staining showed that hindlimb ischemia caused increases of leukocyte infiltration inside myocytes and in the interstitial space in both WT and PD-1^-/-^ mice, and the leukocyte infiltration increased 2.04-fold in PD-1^-/-^ mice as compared with corresponding WT mice (**Figures 2A, B**). Flow cytometry data further demonstrated that hindlimb ischemia resulted in a significantly greater increase of CD45^+^ leukocyte and macrophage infiltration in PD-1^-/-^ mice as compared with the WT mice (**Figure 2D-H**). Macrophages (F4/80^+^CD11b^+^ cells) are the major leukocytes infiltrated in the skeletal muscle in both PD-1^-/-^ and WT mice after ischemia, as the percentages of macrophages in CD45^+^ cells are 86% and 89% in PD-1^-/-^ and WT mice, respectively (**Figure 2F**). In addition, flow cytometry data showed that hindlimb ischemia resulted in a significantly greater increase of CD3+, CD4+, and CD8+ T cell infiltration in PD-1^-/-^ mice as compared with WT mice, although the percentage of these T cells in CD45^+^ leukocytes in PD-1^-/-^ mice was similar to corresponding WT mice (**Figures 2I-Q**). Hindlimb ischemia also resulted in a significantly greater increase of total muscle NK (CD45^+^ CD3^-^NK1.1^+^) cells in PD-1^-/-^ mice as compared with the WT mice (**Figures 2R-T**).

Moreover, hindlimb ischemia caused significant increases of muscle IL-1β, IL-6, MCP-1 and IFN-γ mRNA in both PD-1^-/-^ and WT mice 3 days after femoral artery ligation, while PD-1^-/-^ only caused significantly greater increase of IFN-γ mRNA in mouse skeletal muscles as compared with corresponding WT mice (**Figures 2U, V**) (**Supplemental Figures S3A, B, C**).

### PD-1^-/-^ Enhanced Muscle IFN-γ Production in Mice After Hindlimb Ischemia

Since IFN-γ exerts an important role in regulating PD-1-dependent inflammatory responses, at least in tumor immunology, and since PD-1^-/-^ caused significantly greater increase of muscle IFN-γ mRNA after hindlimb ischemia, we further determined muscle IFN-γ protein content in muscles with or without ischemia from both PD-1^-/-^ and WT mice, and the result demonstrated that PD-1^-/-^ mice significantly increased (1.55 fold) muscle IFN-γ protein in muscle after ischemia (**Figure 2Q**).

### PD-1^-/-^ Exacerbated Muscle Oxidative Stress in Mice After Hindlimb Ischemia

**Figure 3:**
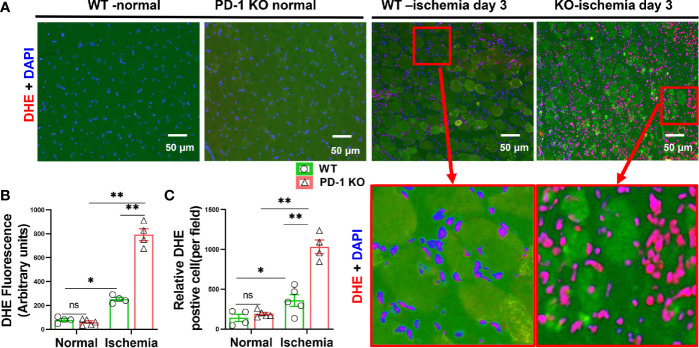
PD-1^-/-^ exacerbated muscle oxidative stress in mice after hindlimb ischemia. **(A)** Representative images of DHE of skeletal muscle from WT and PD-1**^-/-^** mice. Most of the DHE positive cells appear to be the infiltrated leukocytes. **(B, C)** Quantitative data of DHE staining. n=3-5. *P < 0.05 between corresponding groups. **P < 0.01 between corresponding groups. ns, not significant.

We further determined reactive oxidative species (ROS) in gastrocnemius muscle by DHE staining. While the ROS contents of normal muscles were similar in PD-1^-/-^ and WT mice, however, the muscle intracellular ROS production was significantly increased in PD-1^-/-^ mice as compared with WT mice Day 3 after hindlimb ischemia (**Figures 3A-C**). The increase of ROS was predominantly inside the infiltrated leukocytes. The number of ROS positive cells and the ROS signal intensity in these cells were both increased in PD-1^-/-^ mice as compared with WT mice.

### IFN-γ Impaired Vascular Endothelial Proliferation, Migration, and Tube Formation

**Figure 4:**
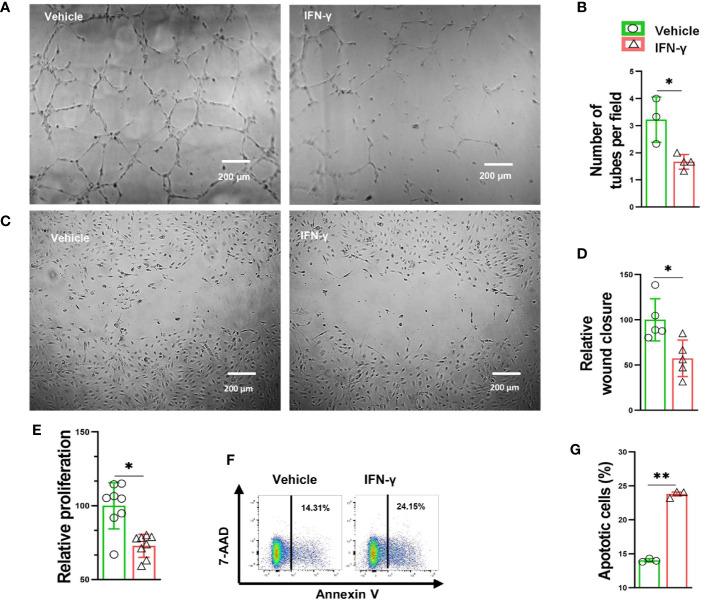
IFN-γ caused HUVEC apoptosis, impaired proliferation and migration, as well as decreased tube formation. **(A)** Representative images of endothelial cell tube formation in response to vehicle (0.1% BSA) or IFN-γ (100U/ml) treatment. **(B)** Quantification of endothelial cell tube formation in response to vehicle or IFN-γ treatment. n=3-4. **(C)** Representative images of endothelial cell migration in response to vehicle or IFN-γ treatment. **(D)** Quantification of endothelial cell migration in response to vehicle or IFN-γ treatment. n=5 **(E)** Quantification of endothelial cell proliferation in response to vehicle or IFN-γ treatment. n=8. **(F, G)** Flow cytometry dot plots and quantification of endothelial cell apoptosis in response to vehicle or IFN-γ treatment. 7-aminoactinomycin D (7-AAD). n=3. Data were compared *via* Student’s t-test. *P < 0.05 between corresponding groups. **P < 0.01 between corresponding groups. ns, not significant.

Since PD-1**^-/-^** caused increased muscle IFN-γ production and inflammation, and attenuated angiogenesis in mice after hindlimb ischemia, we further tested the hypothesis that IFN-γ might regulate angiogenesis through modulating vascular endothelial cell survival and growth capacity. We therefore determined the role of IFN-γ in endothelial apoptosis, proliferation, migration and tube formation. IFN-γ stimulation reduced the vascular tube formation capacity 55% (**Figures 4A, B**). In addition, IFN-γ stimulation also significantly attenuated HUVEC migration 43% as determined by scratch repair assay (**Figures 4C, D**). IFN-γ stimulation also significantly attenuated HUVEC proliferation 28% (**Figure 4E**). Furthermore, we found that IFN-γ caused a significant 70% increase of HUVEC apoptosis (**Figures 4F, G**). These data indicate that IFN-γ can directly regulate angiogenesis through modulating vascular endothelial cell growth, migration, tube formation capacity, and survival.

### PD-1^-/-^ Enhanced Calf Muscle Leukocyte and Macrophage Produced IFN-γ in Mice After Hindlimb Ischemia

**Figure 5:**
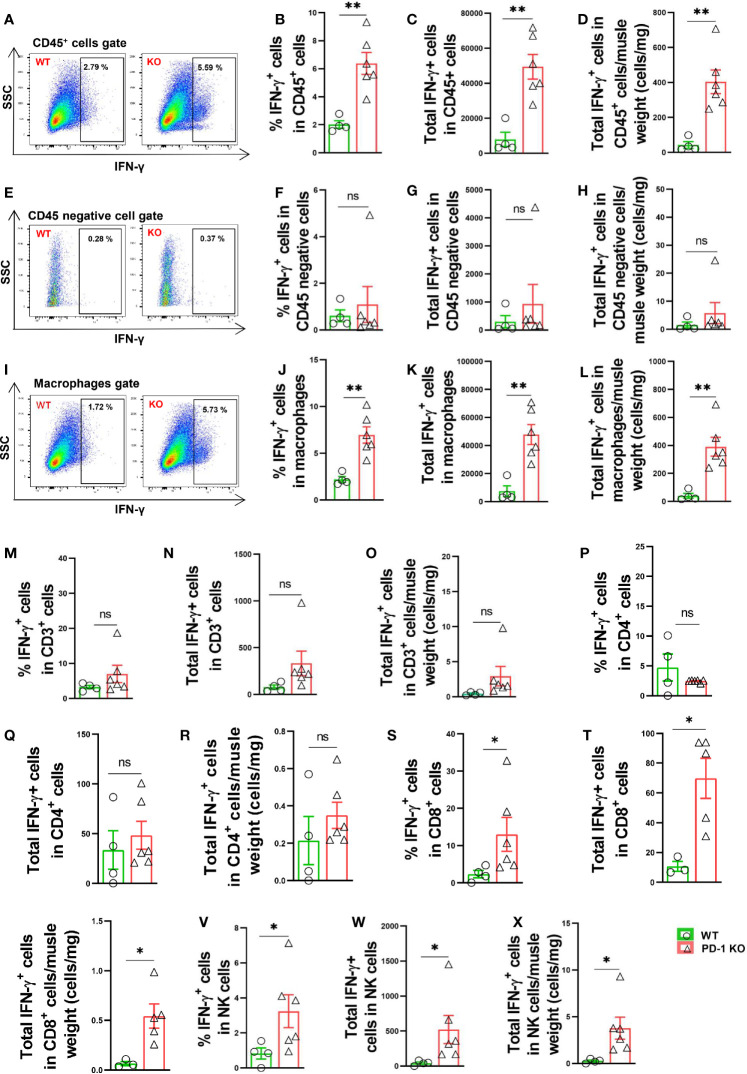
PD-1^-/-^ enhanced muscle leukocyte, NK cell and macrophage produced IFN-γ in mice after hindlimb ischemia. **(A)** Representative IFN-γ^+^ leukocytes (CD45^+^ cells). SSC: Side scatter. **(B–D)** IFN-γ^+^ cells in muscle CD45^+^ cells. n=4-6. **(E)** Representative IFN-γ^+^ cells in muscle CD45 negative cells. **(F–H)** Quantified IFN-γ^+^ cells in muscle CD45 negative gate. n=4-6. **(I)** Representative IFN-γ^+^ macrophages (F4/80^+^CD11b^+^). **(J–L)** Quantified IFN-γ^+^ macrophages. n=4-6. **(M–O)** Quantified IFN-γ^+^ T cells (CD3^+^ T cells). n=4-6. **(P–R)** Quantified IFN-γ^+^ CD4^+^ cells. n=4-6. **(S–U)** Quantified IFN-γ^+^ CD8^+^ cells. n=4-6. **(V–X)** Quantified IFN-γ^+^ NK cells. n=4-6. Data were compared *via* Student’s t-test. *P < 0.05 between groups. **P < 0.01 between groups. ns, not significant.

We further determined IFN-γ production in several leukocyte subsets, and non-leukocytes (CD45 negative cells) in mice 3 days after FA ligation. Interestingly, we found that total IFN-γ positive leukocytes increased 6.2-fold in PD-1^-/-^ mice after hindlimb ischemia, and the percentage of IFN-γ positive leukocytes in CD45^+^ subset also increased 3.0-fold in PD-1^-/-^ mice (**Figures 5A-D**). Meanwhile, the percentage of IFN-γ positive cells in CD45 negative cells (non-leukocytes) were almost undetectable in both WT and PD-1^-/-^ mice (**Figures 5E-H**). As compared with corresponding WT mice, total IFN-γ positive macrophages increased 6.4-fold in PD-1^-/-^ mice after FA ligation, and the percentage of IFN-γ positive macrophages in total macrophages was increased 3.5-fold in PD-1^-/-^ mice after FA ligation (**Figures 5I-L**). Consistent with above findings, the mean signal intensity of IFN-γ were also significantly increased in IFN-γ positive cells in PD-1**^-/-^** mice (**Supplemental Figures S4A-D**). In addition, as compared with corresponding WT mice, the mean signal intensity of IFN-γ positive macrophages in PD-1^-/-^ mice was also significantly increased (**Supplemental Figures S4E, F**). Moreover, as compared with corresponding WT mice, total IFN-γ positive CD3^+^ and CD4^+^ T cells, as well as the percentage of IFN-γ positive cells in CD3^+^ or CD4^+^ T cell subsets were unchanged in PD-1^-/-^ mice after hindlimb ischemia (**Figures 5M-R**). However, total IFN-γ positive CD8^+^ T cells and the percentage of IFN-γ positive CD8^+^ T cells were significantly increased in PD-1**^-/-^** mice after hindlimb ischemia (**Figures 5S-U**). Furthermore, as compared with corresponding mice, total IFN-γ positive NK cells increased 12-fold in PD-1**^-/-^** mice, and the percentage of IFN-γ positive NK cells increased 3.9-fold in PD-1^-/-^ mice after hindlimb ischemia (**Figures 5V-X**).

### PD-1^-/-^ Increased Leukocyte, Macrophage and CD8+ T Cell TNF-α Production in Mice After Hindlimb Ischemia

**Figure 6:**
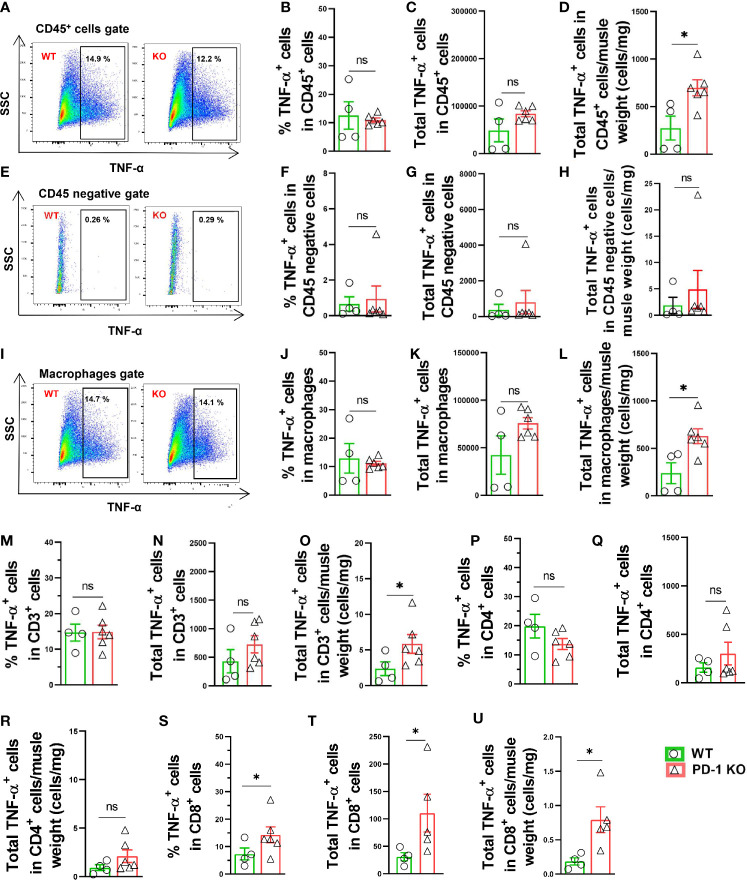
Increase of muscle TNF-α^+^ leukocytes and macrophages in PD-1^-/-^ mice after hindlimb ischemia. **(A)** Representative TNF-α^+^ cells in CD45^+^ cells. SSC: Side scatter. **(B, C, D)** Quantified TNF-α^+^ CD45^+^ cells. n=4-6. **(E)** Representative plots of TNF-α^+^ in CD45 negative cells. **(F–H)** Quantified TNF-α^+^ cells in CD45 negative cells. n=4-6. **(I)** Representative TNF-α^+^ macrophages (F4/80^+^ CD11b^+^). **(J–L)** Quantified TNF-α^+^ macrophages. n=4-6. **(M–O)** Quantified TNF-α^+^ CD3^+^ cells. n=4-6. **(P–R)** Quantified TNF-α^+^ CD4^+^ cells. n=4-6. **(S–U)** Quantified TNF-α^+^ CD8^+^ cells. n=4-6. Data were compared *via* Student’s t-test. *P < 0.05 between groups. ns, not significant.

We also determined the TNF-α production in both CD45^+^ leukocytes and CD45 negative cells in both WT and PD-1^-/-^ mice 3 days after FA ligation. The total numbers of TNF-α positive leukocytes/muscle weight were significantly increased 2.6-fold in PD-1^-/-^ mice after FA ligation, but the percentage of TNF-α positive leukocytes was not increased in PD-1^-/-^ mice (**Figures 6A-D**). The mean signal intensity of TNF-α in leukocytes was also unchanged in PD-1^-/-^ mice (**Supplemental Figures S6A, B**). Meanwhile, the percentage of TNF-α positive cells in CD45 negative cells was almost undetectable in both WT and PD-1^-/-^ mice (**Figures 6E-H**). The mean signal intensity of TNF-α in CD45 negative cells was unchanged in PD-1^-/-^ mice (**Supplemental Figure S6C, D**). The total number of TNF-α positive macrophages/muscle weight in PD-1^-/-^ mice was significantly increased after hindlimb ischemia, but the percentage of TNF-α positive macrophages was not increased in PD-1^-/-^ mice (**Figures 6I-L**). The mean signal intensity of TNF-α positive macrophages in PD-1^-/-^ mice was also unchanged (**Supplemental Figures S6E, F**). Together, these data indicate that the increased muscle TNF-α^+^ leukocytes and macrophages in PD-1^-/-^ mice are the outcome of increased total leukocytes and macrophages in these mice. As compared with corresponding WT mice, total TNF-α positive CD3^+^ and CD4^+^ T cells, as well as the percentage of TNF-α positive cells in CD3^+^ or CD4^+^ T cell subsets were unchanged in PD-1^-/-^ mice after hindlimb ischemia (**Figures 6M-R**). Total TNF-α positive CD8^+^ T cells and the percentage of TNF-α positive CD8^+^ T cells were significantly increased in PD-1**^-/-^** mice after hindlimb ischemia (**Figures 6S-U**).

In addition, we also determined IL10^+^ macrophages (M2 like macrophages), TNF-α^+^ and IFN-γ^+^ macrophages (M1 like macrophages), and the ratios of TNF-α^+^ and IFN-γ^+^ macrophages to IL10^+^ macrophages. While percentage of IL10^+^ macrophages was unchanged, but percentages of TNF-α^+^ and IFN-γ^+^ macrophages were increased in PD-1**^-/-^** mice after hindlimb ischemia (**Supplemental Figures S5A, B**). The ratio of IL10^+^ macrophages to IFN-γ^+^ macrophages were significantly decreased in PD-1**^-/-^** mice after hindlimb ischemia (**Supplemental Figure S5C**). These findings suggest that macrophage polarization was enhanced in PD-1**^-/-^** mice after hindlimb ischemia.

### PD-1 Blocking Antibodies Impaired Blood Flow Recovery, Angiogenesis, and Exercise Ability in Mice After FA Ligation

**Figure 7:**
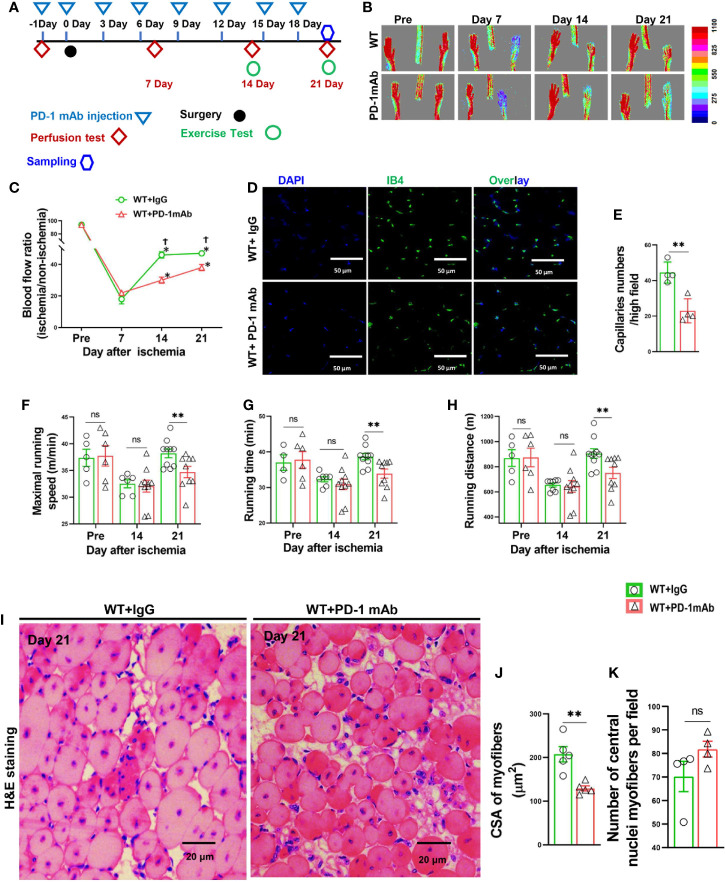
PD-1 mAb treatment impaired blood perfusion, angiogenesis, exercise capacity and skeletal muscle regeneration in mice after hindlimb ischemia. **(A)** The diagram shows the time of relevant interventions. **(B)** Laser doppler perfusion images of ischemia hind limb and non-ischemia hind limb at day pre, 7, 14 and 21. **(C)** Quantification of hind limb perfusion in WT+IgG and WT+ PD-1 mAb mice. *P<0.05 VS Day 0. ^†^P<0.05 VS PD-1 mAb mice. n=4-8. **(D)** Immunostaining of ischemic gastrocnemius muscle with Isolectin GS-IB4 (IB4) to detect capillaries at day 21 after ischemia (green). **(E)** Quantitative analysis of capillary number per high field in WT and PD-1 mAb mice at day 21 after ischemia. n=4. **(F)** Quantitative analysis of maximal speed by forced treadmill running test. n=5-9. **(G)** Quantitative analysis of maximal running time. n=5-9. **(H)** Quantitative analysis of maximal running distance. n=5-9. **(I)** Representative images of H&E of skeletal muscle from PD-1 mAb or IgG treated WT mice undergoing ischemia (day 21). **(J, K)** Quantified myocyte fiber cross-section areas, and the number of muscle fibers with central nuclei in mice after PD-1 mAb or IgG treated WT mice undergoing ischemia (day 21). n=5. ANOVA followed by a Bonferroni correction post-hoc test was used to test for differences among more than 2 groups. Two groups were compared *via* Student’s t-test. *P < 0.05 between groups. **P < 0.01 between groups. ns, not significant.

Since PD-1^-/-^ profoundly attenuated hindlimb ischemia-induced angiogenesis, and since PD-1 blocking antibodies are often used for cancer treatment, we further determined the effect of PD-1 blocking antibodies on hindlimb ischemia-induced angiogenesis in WT mice (**Figures 7A-E**). We found that inhibition of PD-1 with blocking antibodies had no detectable effect on hindlimb blood perfusion in mice before ischemia, shortly after ischemia, and 7 days after ischemia. PD-1 blocking antibodies significantly attenuated blood perfusion 35% and 20% in mice 14 days and 21 days after FA ligation, respectively (**Figures 7B, C**). In addition, PD-1 blocking antibodies significantly attenuated muscle micro-vessel density at 21 days after hindlimb ischemia (**Figures 7D-E**). PD-1 blocking antibodies significantly reduced the overall exercise capacity in mice 14 and 21 days after hindlimb ischemia (**Figures 7F–H**). PD-1 blocking antibodies also significantly exacerbated muscle dystrophy and muscle fibrosis after hindlimb ischemia (**Figures 7I–K**; **Supplemental Figures S7A, B**).

## Discussion

In the present study, we provide the first direct evidence that PD-1 exerts an important role in regulating ischemia-induced hindlimb angiogenesis. We found that inhibition of PD-1 signaling through PD-1 genetic deficiency or pharmacological blocking antibodies effectively attenuated hindlimb blood perfusion, muscle angiogenesis, and exercise capacity in mice after femoral artery ligation. In addition, we found that PD-1^-/-^ significantly exacerbated ischemia-induced muscle oxidative stress and leukocyte infiltration in mice, which occurred before abnormal angiogenesis. We also found macrophages are the major leukocyte subset infiltrated in muscle after hindlimb ischemia. Furthermore, we demonstrated that PD-1^-/-^ enhanced muscle IFN-γ production in mice after hindlimb ischemia, and leukocyte derived IFN-γ production was significantly increased in PD-1^-/-^ mice after hindlimb ischemia. Moreover, we demonstrated that IFN-γ could directly attenuate vascular endothelial cell proliferation, tube formation and migration *in vitro*. IFN-γ also significantly enhanced vascular endothelial cell apoptosis. Together, these data suggest that PD-1 exerts an important role in attenuating ischemia-induced muscle angiogenesis and blood perfusion through modulating the muscle inflammatory response, and the increased leukocyte-derived IFN-γ might partially contribute to the detrimental effects after PD-1 inhibition.

PD-1 is an immune inhibitory receptor predominantly expressed on T cells, and PD-1 delivers its negative signals by binding to its ligands PD-L1 or PD-L2, which are generally expressed on macrophages, dendritic cells, and B cells. Since inflammation regulates ischemia-induced hindlimb angiogenesis (11–13), and since PD-1 plays an important role in regulating inflammatory responses, the findings that PD-1 exerts a profound role in regulating ischemia-induced angiogenesis and blood perfusion in mice after femoral artery ligation were not totally unanticipated. The findings of the current study are conceptionally consistent with the notion that inflammation plays an important role in ischemia-induced hindlimb angiogenesis. In addition, several previous studies demonstrated that PD-1 attenuates vessel inflammation and injury (26, 29). For example, one study showed that PD-1 inhibition enhanced vasculitis or vessel injury (26), and another study showed that PD-1 inhibition exacerbated atherosclerotic lesion development and vessel inflammation (29). In addition, previous studies showed that PD-1^-/-^ promoted stroke-induced brain tissue inflammation and injury in mice (30), and neuronal inflammatory response after spine cord injury in mice (31). Therefore, the enhanced inflammatory response and reduced angiogenesis after inhibition of PD-1 signaling may contribute to the tissue toxicities after the treatment with PD-1 inhibitors. Particularly, inhibition of PD1 signaling pathway may contribute to the development of various cardiovascular diseases such as myocarditis and cardiomyopathy (25, 26). Meanwhile, a decreased neovascularization may also partially contribute to the overall anti-cancer effect of PD-1 inhibitors. The effect of T cells or T cell activation on muscle angiogenesis appear varied in different studies. For example, two previous studies showed that arteriogenic response to hindlimb ischemia was impaired in both CD4 knockout mice or wild type mice after CD4 depletion by blocking antibodies (7, 32). Meanwhile, previous study also demonstrated that CD4^+^ Th1 cells attenuated blood flow recovery in diet-induced T2D mice, and anti-inflammatory Tregs promote blood flow recovery in diet-induced T2D mice (33). One potential explanation is that a mild or moderate inflammatory response is required for the angiogenesis or blood flow recovery, while a severe inflammatory response will attenuate angiogenesis or blood flow recovery.

Hindlimb ischemia-induced inflammation generally peaks 3 to 7 days after femoral artery ligation, while the muscle angiogenesis and blood perfusion generally start to recover 7 to 21 days after hindlimb ischemia (5, 6, 9, 10). Consistent with the previous reports (5, 6, 9, 10), we found that muscle leukocyte infiltration and muscle IFN-γ production were significantly increased in both WT and PD-1^-/-^ mice 3 days after hindlimb ischemia, and the tissue inflammation was more apparent in PD-1^-/-^ mice. Meanwhile, we found that PD-1^-/-^ (and PD-1 blocking antibodies) significantly attenuated hindlimb blood perfusion, muscle angiogenesis, and exercise capacity in mice after femoral artery ligation. The exacerbated muscle fiber dystrophy and the increased number of muscle fibers with central nuclei in PD-1^-/-^ mice after hindlimb ischemia indicate the impaired injury repair capacity in these mice. Increased muscle energy demand results in an increased hindlimb muscle blood perfusion during exercise, and an impaired muscle blood perfusion and endothelial dysfunction attenuate exercise capacity in mice (34). Therefore, the reduced exercise capacity in PD-1^-/-^ mice after hindlimb ischemia is likely an additive effect of inappropriate hindlimb blood perfusion during exercise, inflammation related muscle soreness, stiffness related to muscle fibrosis, and the muscle dystrophy secondary to the abnormal angiogenesis. Since the increased muscle inflammation occurred days before the abnormal muscle angiogenesis in PD-1^-/-^ mice, PD-1^-/-^ likely caused the abnormal muscle angiogenesis and blood perfusion in these mice through increased muscle inflammation after hindlimb ischemia.

IFN-γ is a sole member of type II interferons that is critical for innate and adaptive immunity against viral and some bacterial infections. Aberrant IFN-γ expression is associated with a number of autoinflammatory and autoimmune diseases (35). The increased IFN-γ^+^ macrophages in PD-1^-/-^ mice 3 days after hindlimb ischemia was not fully anticipated. IFN-γ is produced by NK cells, NK T cells, and effective CD4^+^ and CD8^+^ T cells, as well as professional APCs (such as macrophages, dendritic cells and B cells) (36). Consistent with the reports that IFN-γ is mainly produced in leukocytes, we found that muscle IFN-γ^+^ cells are largely undetectable in CD45 negative cells, and IFN-γ^+^CD45^+^ leukocytes are significantly increased in PD-1^-/-^ mice 3 days after hindlimb ischemia. The dramatic increase of IFN-γ^+^ NK cells and CD8+ T cells and CD8+ T cells in PD-1^-/-^ mice after hindlimb ischemia supports an important role of NK cells and CD8+ T cells in IFN-γ production. IFN-γ^+^ macrophages were significantly increased in PD-1^-/-^ mice 3 days after hindlimb ischemia. The finding of muscle IFN-γ^+^ macrophages is consistent with previous reports that IFN-γ is produced by macrophages in humans and mice (37–39). The increase of TNF-α^+^ and IFN-γ^+^ macrophages (M1 like macrophages), and the ratios to IL10^+^ macrophages (M2 like macrophages) in PD-1**^-/-^** mice after hindlimb ischemia indicate that M1-like macrophage polarization was enhanced in PD-1**^-/-^** mice.

Interestingly, we found that PD-1^-/-^ exacerbated muscle IFN-γ production in mice 3 days after hindlimb ischemia. Since IFN-γ production exerts an important role in regulating tissue inflammation and injury, and since IFN-γ can directly attenuate vascular endothelial cell growth and survival, the increased muscle IFN-γ in PD-1^-/-^ mice might contribute to the increased tissue inflammation and abnormal angiogenesis in these mice after femoral artery ligation. Since previous studies have showed that IFN-γ can directly attenuate skeletal muscle fiber differentiation (40), the increased IFN-γ production may directly attenuate muscle injury repair in PD-1^-/-^ mice after ischemia. Moreover, as PD-1^-/-^ and pharmacological PD-1 inhibition also caused alteration in other inflammation-associated genes, it is almost certain that PD-1 exerts its protective role beyond IFN-γ.

The present study has several major limitations. First, restoration of the blood flow after hindlimb ischemia requires both angiogenesis and arteriogenesis, but the effect of PD-1 inhibition on arteriogenesis was not determined in our study. Second, although the mouse is a commonly used animal model for various pre-clinical research studies, since the clinical ischemic conditions often occur in old patients with various co-morbidities, the findings from the well-controlled young mice may not fully mimic the clinical population. Third, we only studied female mice in the present study. However, ischemia-induced tissue inflammation and angiogenesis are commonly observed in both male and female mice after femoral artery ligation, and since PD-1 regulates inflammation in mice of both genders in various disease models, we anticipate that PD-1 inhibition would have similar impacts on tissue inflammation and angiogenesis after hindlimb ischemia in both sexes. Finally, PD-1 regulate T cell activation, and activated T cells show increased expressions of CD44, CD69, and reduced expression CCR7 and CD62L. Above changes often contribute to the increased proinflammatory cytokine production. However, due to the limited number of infiltrated T cells in muscle tissues, the T cell activation markers were not detected.

## Conclusion

Our findings indicate that PD-1 exerts an important role in regulating ischemia-induced angiogenesis through modulating the tissue inflammatory response.

## Data Availability Statement

The datasets presented in this study can be found in online repositories. The names of the repository/repositories and accession number(s) can be found in the article/**Supplementary Material**.

## Ethics Statement

The animal study was reviewed and approved by Animal Care and Use Committee of Shanghai University of Sport, China.

## Author Contributions

YC and PC designed research and wrote the manuscript. XL performed research, analyzed data and wrote the manuscript. XW, WX and XX performed research. All authors contributed to the article and approved the submitted version.

## Funding

This study was supported by the Key Lab of Development and Protection of Human Sports Ability in Shanghai (No. 11DZ2261100), China scholarship council (No. 201808310117), China Postdoctoral Science Foundation (2019M661374).

## Conflict of Interest

The authors declare that the research was conducted in the absence of any commercial or financial relationships that could be construed as a potential conflict of interest.
