# Supplementary material for: Pharmacological and Genetic Inhibition of PD-1 Demonstrate an Important Role of PD-1 in Ischemia-Induced Skeletal Muscle Inflammation, Oxidative Stress, and Angiogenesis
Source: Front Immunol. 2021 Mar 19;12:586429. doi: 10.3389/fimmu.2021.586429 (PMC8017157; doi:10.3389/fimmu.2021.586429)
Supplement: Supplementary file 1 [file DataSheet_1.docx]

**Supplemental File**

**Pharmacological and genetic inhibition of PD-1 demonstrate an important role of PD-1 in ischemia-induced skeletal muscle inflammation, oxidative stress, and angiogenesis**

**Running title:** PD-1 and hindlimb ischemia-induced angiogenesis

Xiaoguang Liu^1^, Xinyu Weng^2,3^, Weihua Xiao^4^, Xin Xu^4^, Yingjie Chen^2,5*^, Peijie Chen^4**^

^1^ College of Sports and Health, Guangzhou Sport University, Guangzhou 510500, China

^2^Lillehei Heart Institute and Cardiovascular Division, University of Minnesota Medical School, Minneapolis, MN 55455, USA

^3^Department of Cardiology, Zhongshan Hospital, Fudan University. Shanghai Institute of Cardiovascular Diseases, Shanghai 200032, China

^4^School of Kinesiology, Shanghai University of Sport, Shanghai 200438, China

^5^Department of Physiology & Biophysics, University of Mississippi Medical Center, Jackson, MS 39216, USA

Address for correspondence: Yingjie Chen, MD, PhD

email: [ychen2@umc.edu](mailto:ychen2@umc.edu)

Peijie Chen, Ph.D.

email: chenpeijie@sus.edu.cn

**Supplementary figures/tables**


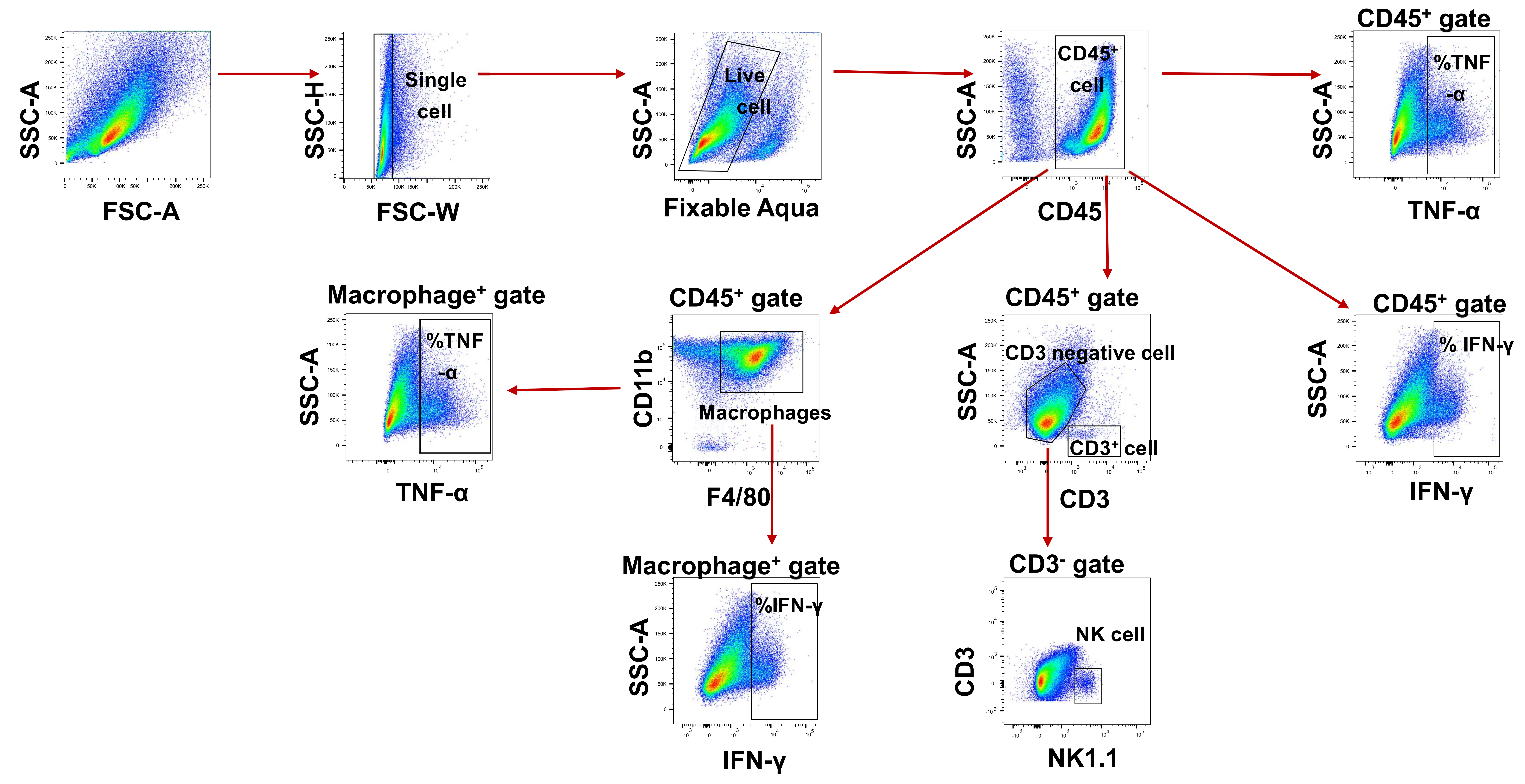


**Figure. S1. The gating strategy used for flow cytometry.**


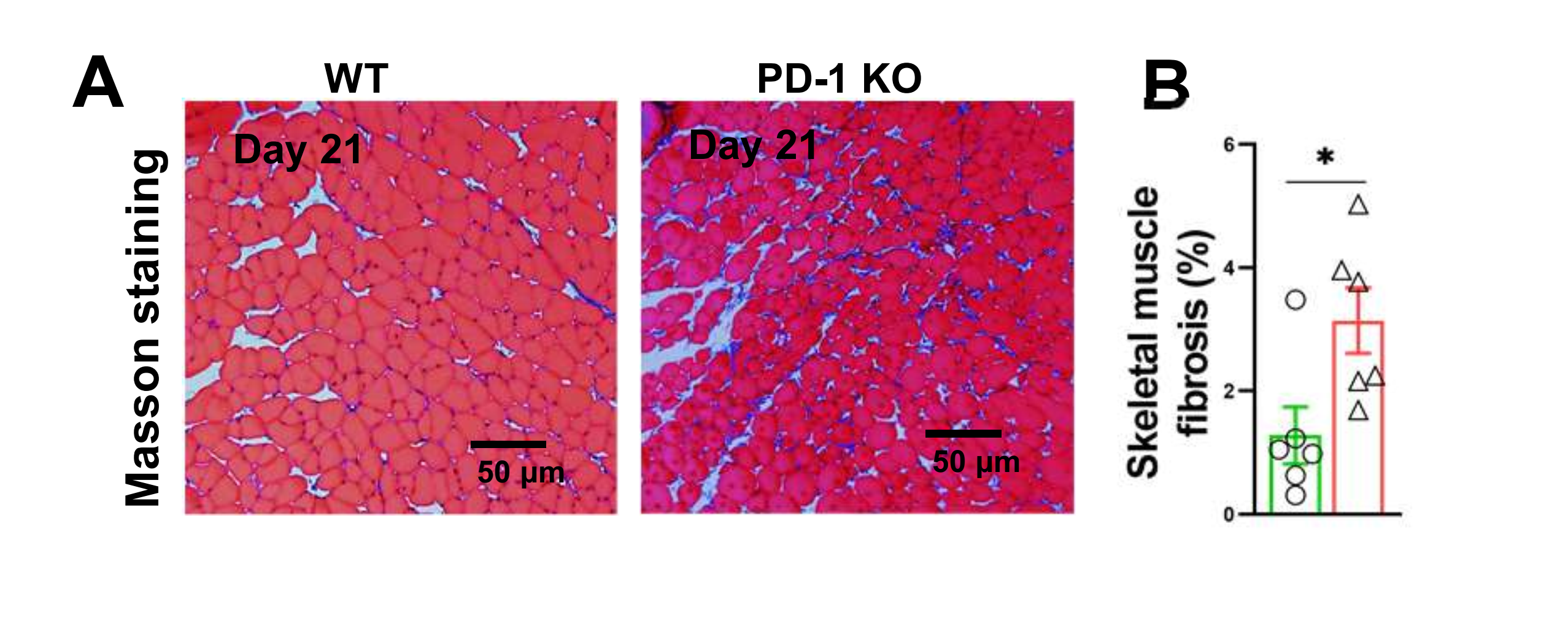


**Figure. S2. PD-1^-/-^ exacerbated mouse muscle fibrosis after hindlimb ischemia. (A)** Representative images of Masson staining of skeletal muscle from WT and PD-1^-/-^ mice undergoing ischemia (day 21). **(B)** Quantification of fibrosis from WT and PD-1^-/-^ mice undergoing ischemia (day 21). n=6. Data were compared via Student’s t-test. *P<0.05 between corresponding groups. **P<0.01 between corresponding groups. ns, non-significant. Data are mean ± SEM.


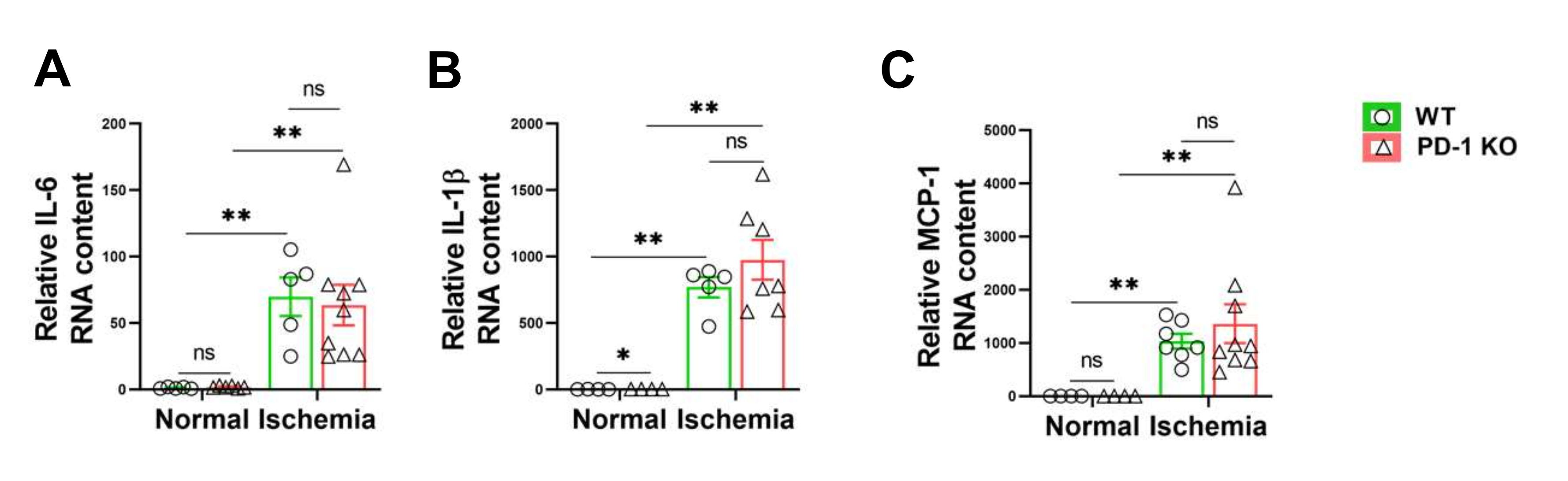


**Figure. S3. PD-1^-/-^ exacerbated muscle inflammation in mice after hindlimb ischemia. (A-C)** Quantitative reverse-transcriptase polymerase chain reaction (RT-PCR) results of IL-6, IL-1β and MCP-1 in skeletal muscle. n=4-7**.** Two-way ANOVA followed by a Bonferroni correction post-hoc test was used to test for differences among more than 2 groups. *P<0.05 between corresponding groups. **P<0.01 between corresponding groups. ns, non-significant. Data are mean ± SEM.


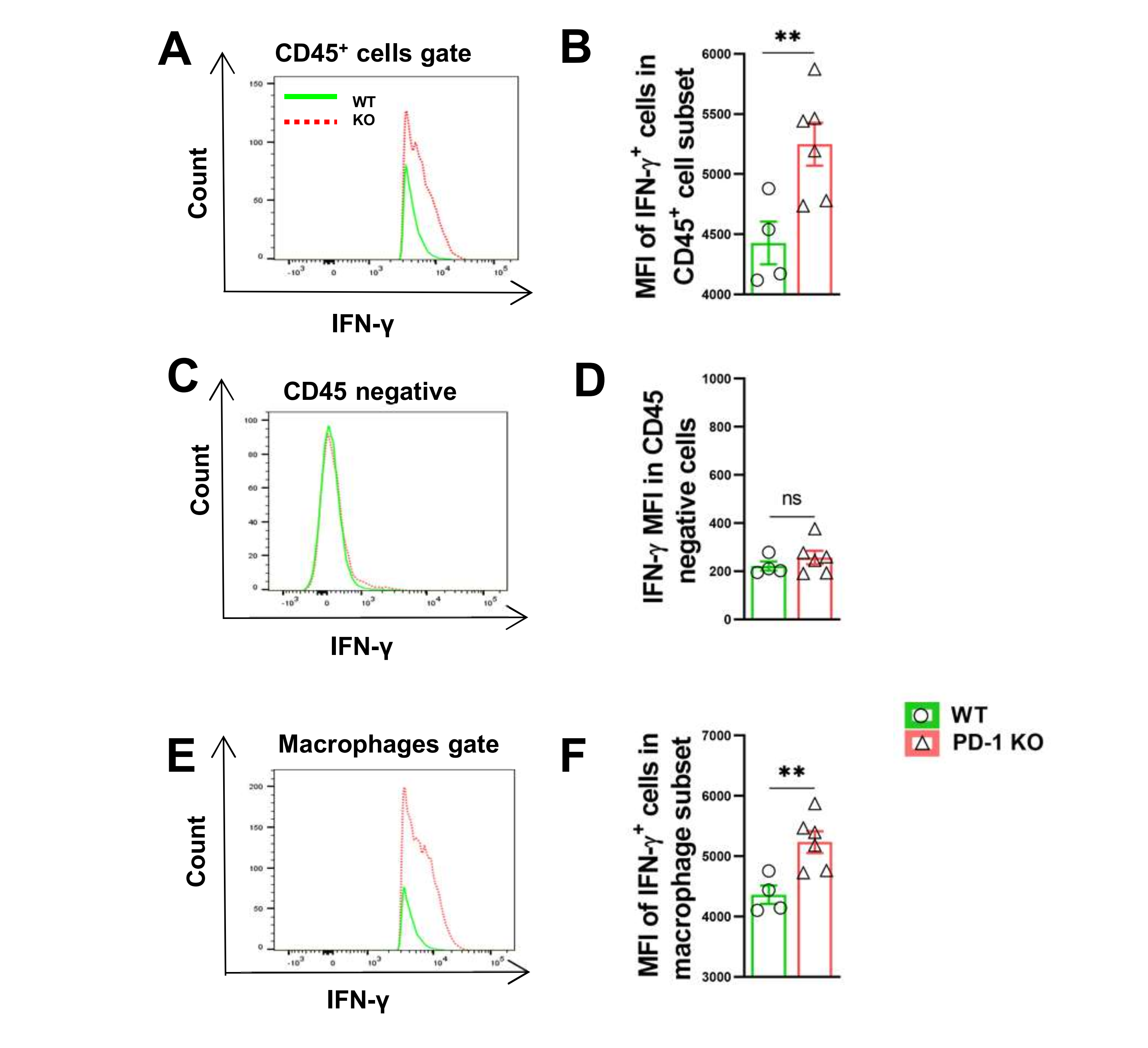


**Figure. S4. PD-1^-/-^ enhanced muscle leukocyte cell produced IFN-γ in mice after hindlimb ischemia. (A)** Representative flow cytometry histograms of fluorescence intensity of IFN-γ^+^ cells in muscle CD45^+^ gate. **(B)** Quantification of mean fluorescence intensity (MFI) of IFN-γ^+^ cells in muscle CD45^+^ gate. n=4-6**. (C)** Representative flow cytometry histograms of IFN-γ^+^ cells fluorescence intensity in muscle CD45 negative gate.  **(D)** Quantification of IFN-γ^+^ cells mean fluorescence intensity (MFI) in muscle CD45 negative gate. n=4-6**. (E)** Representative flow cytometry histograms of fluorescence intensity of IFN-γ^+^ cells in muscle macrophage gate. n=4-6**. (F)** Quantification of mean fluorescence intensity (MFI) of IFN-γ^+^ cells in muscle macrophage gate. n=4-6**.** Data were compared via Student’s t-test. *P<0.05 between corresponding groups. **P<0.01 between corresponding groups. ns, non-significant. Data are mean ± SEM


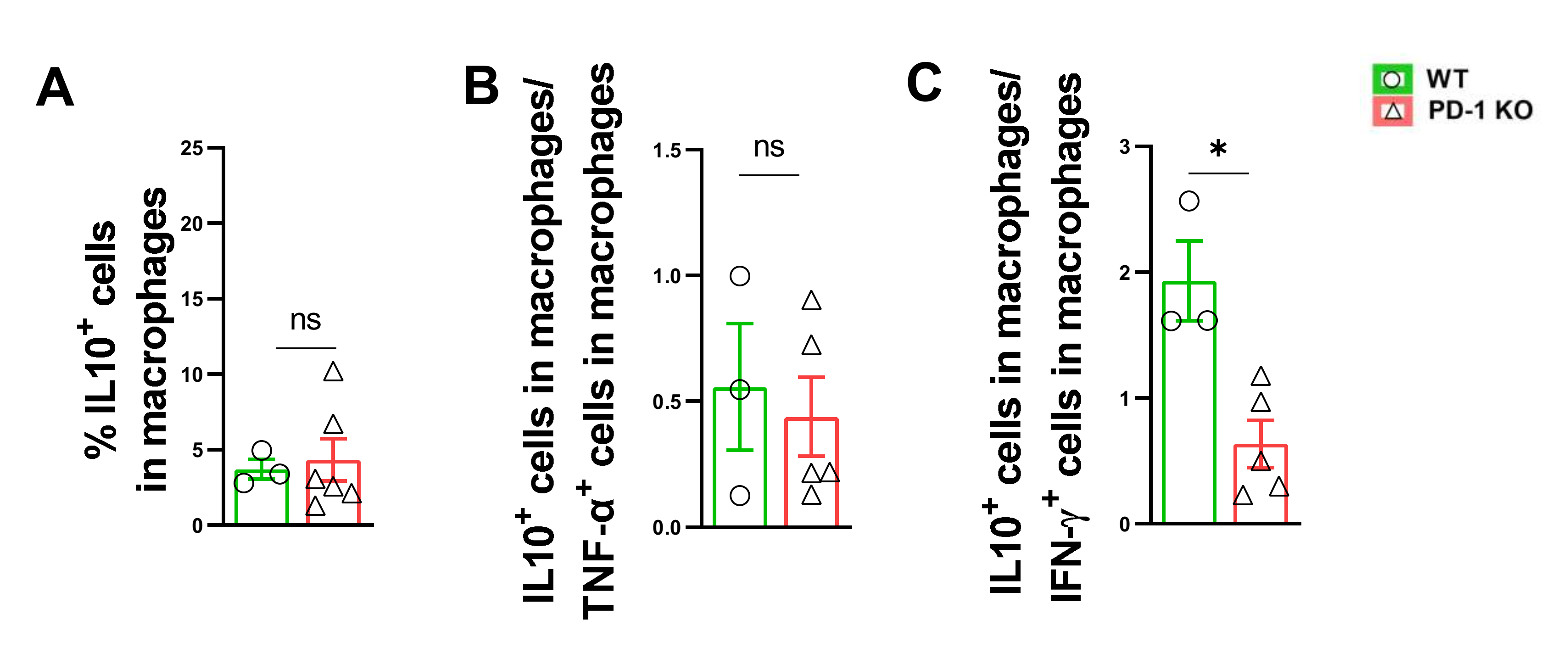


**Figure. S5. Decrease of muscle IL-10^+^ macrophages / IFN-γ^+^ macrophages in PD-1^-/-^ mice after hindlimb ischemia. (A)** Quantified IL-10^+^ macrophages. **(B)** Quantified IL-10^+^ macrophages/ TNF-α^+^ macrophages. **(C)** Quantified IL-10^+^ macrophages/IFN-γ^+^ macrophages.


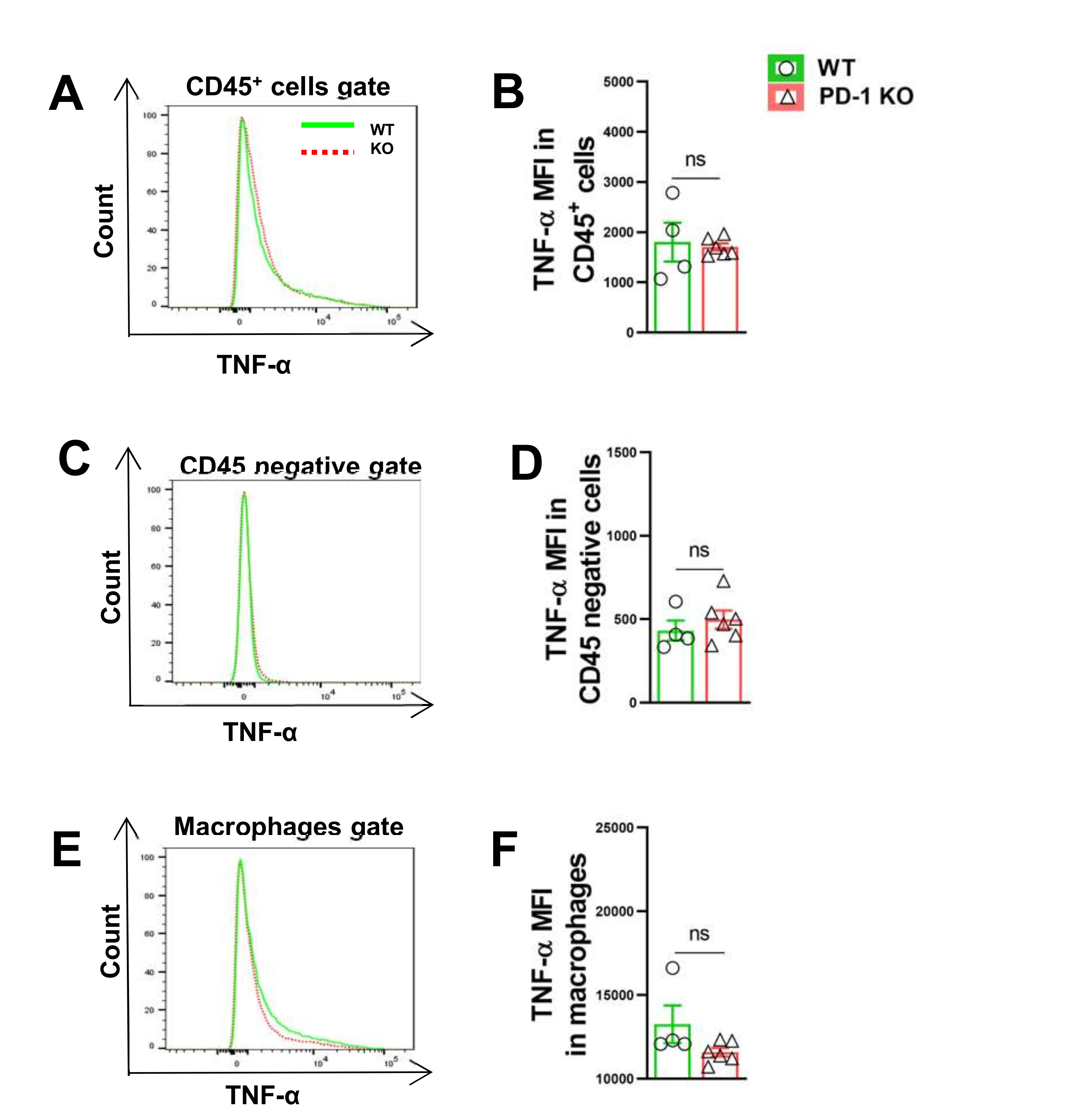


**Figure. S6. Increase of muscle TNF-α^+^ leukocytes and macrophages in PD-1^-/-^ mice after hindlimb ischemia. (A)** Representative flow cytometry histograms of TNF-α^+^ cells fluorescence intensity in muscle CD45^+^ gate. **(B)** Quantification of TNF-α^+^ cells mean fluorescence intensity (MFI) in muscle CD45^+^ gate. n=4-6**. (C)** Representative flow cytometry histograms of TNF-α^+^ cells fluorescence intensity in muscle CD45 negative gate.  **(D)** Quantification of TNF-α^+^ cells mean fluorescence intensity (MFI) in muscle CD45 negative gate. **(E)** Representative flow cytometry histograms of TNF-α^+^ cells fluorescence intensity in muscle macrophage gate (F4/80^+^ CD11b^+^). **(F)** Quantification of TNF-α^+^ cells mean fluorescence intensity (MFI) in muscle macrophage gate. n=4-6**.** Data were compared via Student’s t-test. *P<0.05 between corresponding groups. **P<0.01 between corresponding groups. ns, non-significant. Data are mean ± SEM.


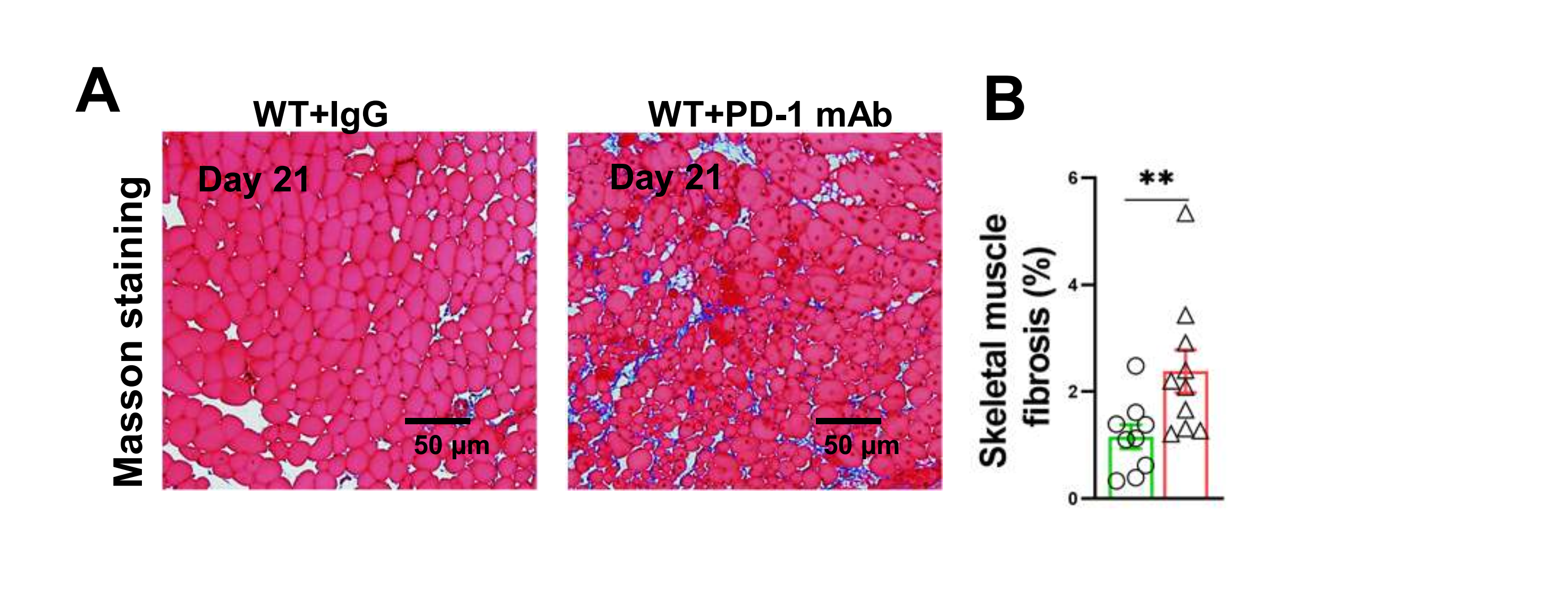


**Figure. S7. PD-1 mAb treatment exacerbated mouse muscle fibrosis after hindlimb ischemia. (A)** Representative images of Masson staining of skeletal muscle from PD-1 mAb or IgG treated WT mice undergoing ischemia (day 21). **(B)** Quantification of fibrosis from PD-1 mAb or IgG treated WT mice undergoing ischemia (day 21). n=10. Data were compared via Student’s t-test. *P<0.05 between corresponding groups. **P<0.01 between corresponding groups. ns, non-significant. Data are mean ± SEM.

**Table S1. Primary antibodies used in our study**

| **Antibody** | **Clone** | **Name of the company** | **Catalog number** |
| --- | --- | --- | --- |
| anti-mouse CD16/32 | 2.4G2 | BD Biosciences | 553142 |
| AF700-conjugated anti-CD45 | 104 | eBioscience | 56045482 |
| BUV737-conjugated anti-CD3 | 145-2C11 | BD Biosciences | 564618 |
| BUV395-conjugated anti-CD4 | RM4-5 | BD Biosciences | 740208 |
| BUV805-conjugated anti-CD8 | 53-6.7 | BD Biosciences | 612898 |
| PE/CY7-conjugated anti-CD11b | M1/70 | eBioscience | 25011282 |
| BV785-conjugated anti-F4/80 | BM8 | Bioglegend | 123141 |
| APC-conjugated anti-NK1.1 | PK136 | eBioscience | 17594182 |
| BV650-conjugated anti-TNF-α | MP6-XT22 | Bioglegend | 506333 |
| BV711-conjugated anti-IFN-γ | XMG1.2 | BD Biosciences | 564336 |
| CD45 |  | R&D systems | AF114 |
| AF488-conjugated Isolectin GS-IB4 (IB4) |  | Invitrogen | I21411 |
| Dihydroethidium (Hydroethidine) |  | Invitrogen | DS23107 |
| In vivo mAb PD-1 | J43 | Bioxcell | BP0033-2 |

**Table S2 Primers Used for RT-PCR**

| Target gene | Primer sequences |  |
| --- | --- | --- |
| IL-6 | 5’-GAACAACGATGATGCACTTGC-3’ | |
|  | 5’-CTTCATGTACTCCAGGTAGCTATGGT-3’ | |
| MCP-1 | 5’-GCTCAGCCAGATGCAGTTAAC-3’ | |
|  | 5’- CTCTCTCTTGAGCTTGGTGAC-3’ | |
| GAPDH | 5’-ACTCCACTCACGGCAAATTC-3’ | |
|  | 5’-TCTCCATGGTGGTGAAGACA-3’ | |
| IL-1β | 5’-TGACGTTCCCATTAGACAACTG -3’ | |
|  | 5’-CCGTCTTTCATTACACAGGACA-3’ | |
| INF-γ | 5’-GCTTTGCAGCTCTTCCTCAT -3’ | |
|  | 5’-GTC ACC ATCCTTTTGCCAGT -3’ | |

**Materials and methods**

**Hindlimb ischemia model and laser-Doppler perfusion imaging:** The mice were anesthetized using 2% isoflurane, and the hair from both hindlimbs was shaved with a small animal electric trimmer (Shenzhen Ruiwode Life Technology Co., Ltd). The femoral artery (FA) was exposed by incision of the skin at the middle portion of the left hindlimb. Both the proximal end and the distal end of the FA were ligated and the FA between the ligatures was excised. The incision was then closed by interrupted 5-O Vicryl sutures ^1, 2^.

Shortly after surgery, as well as 3, 7, 14, and 21days postoperatively, mice were placed on a heating pad for several minutes and the blood flow of both hindlimbs was determined with a high-resolution laser doppler imager (MOORLDI2-HIR system, Moor instruments, UK). Colour-coded images were obtained with a Moor laser-Doppler imager (LDI) scanner. The perfusion of each mouse was calculated by the Moor LDI software [region of interests (ROIs). The perfusion of the ischemia limb is expressed as a percentage of the perfusion to the non-ischemic limb ^3^.

**Determination of mouse exercise capacity:** Mice were pretrained on the treadmill twice (at a slow speed 10-15 m/min for 10 min with 7° grade) one week before hindlimb ischemia surgery. On each day of testing, the initial speed was 12.5 m/min and the speed was increased up to 15m/min, at a rate of 0.5m/min. After that, the speed was further increased every 3 minutes at a rate of 2.5 m/min until the mice was unable to maintain the required running speed (demonstrated by receiving electric shocks without attempts to run). The maximum speed, running time, and total running distance were recorded in each mouse ^4^.

**Histological staining:** Gastrocnemius muscle was excised, fixed in 10% formalin, and then embedded in paraffin. 7 μm thick sections were prepared and used for H&E staining, and Masson’s Trichrome staining as previously described ^5, 6^.The capillary and micro-vessels were determined by staining of Isolectin GS-IB4 (IB4) using Alexa Fluor™ 488 Conjugate antibody (Invitrogen, California, United States). These data were measured and calculated using Image-J software.

**Immunofluorescence staining for leukocyte infiltration:** For immunofluorescence staining, 7μm- thick gastrocnemius muscle sections were deparaffined, rehydrated with gradient ethanol, then treated with citrate buffer (pH = 6.0) for antigen retrieval and blocked with serum for 1h at room temperature. This was followed by incubation with primary antibodies at 4°C overnight. The primary antibodies utilized for these experiments were CD45 (R&D systems, 1:100). Alexa Fluor 555-conjugated donkey anti-goat antibody (1:500) (Invitrogen, United States) was used as the secondary antibody for CD45. Imaging was performed on an OLYMPUS BX53 microscope.

**Intracellular ROS measurement:** Gastrocnemius muscle was immediately embedded in an optimum cutting temperature compound (OCT) and stored at -80^o^C. Dihydroethidium (DHE) (Invitrogen, cat: D23107) (5μmol/L) was applied to 10 μm fresh frozen tissue sections, incubated in the light-protected humidified chamber at 37°C for 30 min, washed with PBS three times, 5 minutes per wash, and then the sections were cover-slipped using Mounting Medium with DAPI (abcam, ab104139). Imaging was performed using a fluorescence microscope (ZEISS Axio Imager 2).

**Enzyme-linked immunosorbent assay (ELISA):** Gastrocnemius muscle IFN-γ levels were measured in homogenized skeletal muscle using a mouse IFN-γ ELISA kit from Invitrogen (cat：BMS606) according the manufacturer’s instructions. The detection limit of this ELISA Kit for IFN-γ is 5.3 pg/ml.

**Detection of leukocyte intracellular cytokine production:** The tibial muscle and gastrocnemius were collected and weighed. Tissues were cut into ~2x2x2 mm segments using a sterilized scissors, then digested in PBS buffer with collagenase II (2 mg/ml, Invitrogen) and DNase I (150 Ug/ml, Sigma) for 40 min. Digested muscle was filtered through a 70 µm cell strainer. To enrich the leukocytes from the digested muscle, the isolated cells were resuspended in 40% Percoll (Sigma-Aldrich) overlaid on 80% Percoll, then centrifuged at 400 g for 25 min. The interphase that contains leukocytes was collected, washed and stored in Flow Cytometry Staining Buffer (Invitrogen, California, United States) for detection of intracellular cytokine production.

Briefly, leukocytes were suspended in Staining Buffer (Invitrogen, California, United States), stimulated with Cell Stimulation Cocktail (Phorbol 12-Myristate 13-Acetate (40.5 µM), lonomycin (670 µM), Brefedin A (5.3 mM), Monensin (1 mM) in Ethanol (500X)) (Invitrogen, California, United States) for 5 h at 37℃ in completed RPMI-1640 media (Invitrogen, California, United States). Cell suspensions were stained with Zombie Aqua™ Fixable Viability Kit (Biolegend, California, United States) and antibodies against CD45 (104), F4/80 (BM8), TNF-alpha ( MP6-XT22), all from Biolegend (California, United States), CD3 (145-2C11), IFN-gamma ( XMG1.2) from BD Biosciences (California, United States), and CD11b (M1/70), from (eBioscience, California, United States). Samples were then processed detecting corresponding leukocyte subsets and cytokine productions using a flow cytometer (BD LSRFortessa™ X-20, United States). Data were analyzed by FlowJo software version 10 (FlowJo LLC, United States) ^7-9^.

**HUVEC cell culture****:** Primary human umbilical artery endothelial cells (HUVEC) (LONZA, Basel, Switzerland) were maintained in EGM medium (Cambrex, New Jersey, United States) under 5% CO2 in a humidified incubator at 37 °C.  Cells at passage 4 to 6 were used for these experiments.

**Cell proliferation assay:** HUVEC cell proliferation was assayed by CyQUANT® Cell Proliferation Assay Kit (Invitrogen, Cat: CT206) after IFN-γ (Biolegend, cat: 713906) (100U/ml) or vehicle (0.1% bovine serum albumin (BSA)) treatment for 24 hours.

**Vascular endothelial cell tube formation assay:** Tube formation capacity was determined using an in vitro angiogenesis assay kit from R&D Systems (cat: 3470-096-K). Briefly, HUVEC were overlaid onto 15 µl Matrigel (BD Biosciences, California, United States) in 15-well plates (ibidi, Cat: 81506) treated with either IFN-γ (100U/ml) or vehicle (0.1% BSA) for 4 hours. After being washed by PBS, digital photographs were then obtained.  Each experiment was performed in triplicate, and 5 random pictures were taken of each well at a magnification of 10X.  Linear “tube” formation was quantified using the method described by Zhang et al ^10^.

**Vascular endothelial cell migration test:** To assay the endothelial cell migration, HUVEC were seeded at 1x10^5^ cells per well (6-well plate) one day before INF- γ treatment. After that, the HUVEC layer was scratched across the culture dish by a 200 μl sterile pipet tip and washed with 1x PBS. After IFN-γ (100U/ml) or vehicle (0.1% BSA) treatment for 24 h, photos were taken by an inverted microscope (ZEISS Axio Vert.A1). The results were measured by ImageJ Software and presented as the percentage of scratch filled by the HUVEC ^11^.

**Vascular endothelial cell apoptosis assay:** Cell apoptosis was determined using FITC Annexin V Apoptosis Detection Kit with 7-aminoactinomycin D (7-AAD) (Biogend, cat: 640922). Briefly, 1x10^5^ cells per well were seeded on 6-well plates for 24 hours. After that, media containing IFN-γ (100U/ml) or vehicle (0.1% BSA) were used to treat for 24 hours, cells were washed with cell-staining buffer and resuspended in 100 μl of cell-staining buffer. The FITC Annexin V and 7-AAD were added in the HUVEC cell suspension and incubated for 15 min at room temperature in the light-protected condition. Then 400 μl of Annexin V Buffer was added to each sample and tested by flow cytometer (BD FACS AriaII, United States). Data were analyzed by FlowJo software version 10 (FlowJo LLC, United States).

**References:**

1. Niiyama H, Huang NF, Rollins MD, Cooke JP. Murine model of hindlimb ischemia. *Journal of visualized experiments : JoVE* 2009.

2. Padgett ME, McCord TJ, McClung JM, Kontos CD. Methods for Acute and Subacute Murine Hindlimb Ischemia. *Journal of visualized experiments : JoVE* 2016.

3. Sharir R, Semo J, Shaish A, Landa-Rouben N, Entin-Meer M, Keren G, George J. Regulatory T cells influence blood flow recovery in experimental hindlimb ischaemia in an IL-10-dependent manner. *Cardiovasc Res* 2014;103:585-596.

4. Jordan SD, Kriebs A, Vaughan M, Duglan D, Fan W, Henriksson E, Huber AL, Papp SJ, Nguyen M, Afetian M, Downes M, Yu RT, Kralli A, Evans RM, Lamia KA. CRY1/2 Selectively Repress PPARdelta and Limit Exercise Capacity. *Cell metabolism* 2017;26:243-255 e246.

5. Liu X, Zhen L, Zhou Y, Chen Y, Chen P, Xiao W. BMSC Transplantation Aggravates Inflammation, Oxidative Stress, and Fibrosis and Impairs Skeletal Muscle Regeneration. *Frontiers in physiology* 2019;10:87.

6. Liu X, Zeng Z, Zhao L, Chen P, Xiao W. Impaired Skeletal Muscle Regeneration Induced by Macrophage Depletion Could Be Partly Ameliorated by MGF Injection. *Frontiers in physiology* 2019;10:601.

7. Kuswanto W, Burzyn D, Panduro M, Wang KK, Jang YC, Wagers AJ, Benoist C, Mathis D. Poor Repair of Skeletal Muscle in Aging Mice Reflects a Defect in Local, Interleukin-33-Dependent Accumulation of Regulatory T Cells. *Immunity* 2016;44:355-367.

8. Panduro M, Benoist C, Mathis D. Treg cells limit IFN-gamma production to control macrophage accrual and phenotype during skeletal muscle regeneration. *Proceedings of the National Academy of Sciences of the United States of America* 2018;115:E2585-E2593.

9. Burzyn D, Kuswanto W, Kolodin D, Shadrach JL, Cerletti M, Jang Y, Sefik E, Tan TG, Wagers AJ, Benoist C, Mathis D. A special population of regulatory T cells potentiates muscle repair. *Cell* 2013;155:1282-1295.

10. Zhang P, Hu X, Xu X, Chen Y, Bache RJ. Dimethylarginine dimethylaminohydrolase 1 modulates endothelial cell growth through nitric oxide and Akt. *Arteriosclerosis, thrombosis, and vascular biology* 2011;31:890-897.

11. Salmeron K, Aihara T, Redondo-Castro E, Pinteaux E, Bix G. IL-1alpha induces angiogenesis in brain endothelial cells in vitro: implications for brain angiogenesis after acute injury. *J Neurochem* 2016;136:573-580.
